# Promoting supportive strategies to enable nursing students to manage anxiety during their training

**DOI:** 10.4102/curationis.v49i1.2866

**Published:** 2026-09-04

**Authors:** Hlengiwe D. Seshabela, Simangele Shakwane

**Affiliations:** 1 Department of Health Studies, College of Human Sciences, University of South Africa, Pretoria, South Africa; 2 Department of Health, Gauteng College of Nursing, Pretoria, South Africa

**Keywords:** anxiety-related conditions, nursing students, promoting, supportive strategies, manage and training

## Abstract

**Background:**

Anxiety is highly prevalent among nursing students, even in normal circumstances. This is because of the demanding academic workload, which includes both theoretical and work-integrated learning, as well as caring for ill patients and working shifts. Nursing students experience anxiety as a result of different factors and employ various coping mechanisms to manage their responses.

**Objective:**

To develop and validate supportive strategies that will enable nursing students to manage their anxiety during training.

**Method:**

A qualitative descriptive phenomenological design was employed. This approach enabled the researcher to explore and describe the lived experiences of nursing students and the perspectives of nurse educators regarding students’ anxieties. The study was conducted in two phases. In phase 1, an empirical study was conducted through semi-structured interviews and focus group discussions; data were analysed using a phenomenological method. In phase 2, supportive strategies for managing students’ anxiety during training were developed and validated using the World Health Organization’s (2018) guidelines.

**Results:**

The integrated results from phase 1 are presented as a summary of findings. Both groups revealed a limited understanding of anxiety-related conditions. New students are not assessed for mental health, and supportive resources are lacking.

**Conclusion:**

Supportive strategies are essential to enable nursing students to manage their anxiety in an attempt to promote mental well-being during training.

**Contribution:**

Developed and validated supportive strategies to manage anxiety during training, which were paramount to increasing retention rate, promoting academic success and mental well-being of nursing students.

## Introduction

Nursing education programmes include theoretical, clinical and psychosocial components, which can make nursing students more susceptible to anxiety-related conditions (Savitsky et al. 2020:1). Nursing programmes are the most stressful and emotionally draining profession, with demanding theoretical and work-integrated learning (Manana et al. 2023:1). Research shows that anxiety-related disorders are more common among nursing students than in the general population (Otim et al. 2021:1863). Anxiety-related conditions are the most common mental disorders globally, with a current prevalence of 7.3% (Stein et al. 2021:336). Anxiety is an uneasy feeling that manifests as a non-adaptive physical and psychological reaction when individuals encounter intrusive thoughts and uncertainty (Xi 2020:10). Anxiety disorder is a chronic emotional state that is unpleasant and affects psychological and physiological aspects, including obsessive-compulsive, trauma and stress-related disorders (Keltner & Steele 2023:316). This state of anxiety is characterised by discomfort and worry caused by various stressors and manifests through both physical and psychological symptoms (Middleton 2020:288). Anxiety-related conditions are rooted in stress and encompass disorders like obsessive-compulsive disorder, trauma and specific phobias (Macintyre, Zare & Williams 2023:31). Several factors contribute to anxiety in nursing students, including financial constraints, academic pressures and the need to balance multiple responsibilities (Jacobs, Scrooby & Du Preez 2019:6).

Anxiety can impact nursing students’ motivation to learn, academic effectiveness and even their willingness to pursue a nursing career (Garcia-Gonzalez et al. 2021:8). Promotion, of strategies, prevention and early intervention strategies may produce the greatest impact on individuals’ mental health and well-being (Colizzi, Lasalvia & Ruggeri 2020:2). Jamil et al. (2022:112) assert that there is a negative relationship between anxiety and academic performance, highlighting the importance of prevention efforts to reduce student attrition.

In South Africa, nursing students face a high failure rate, which leads to demotivation and dropout (Savitsky et al. 2020:1). The study of Mudaly (2023:2) shows that 22% of nursing students leave the programme after the first year of study. Among nursing students, 37.1% experience anxiety disorders, while 38.7% have disruptive behaviours, making these disorders more common than other types of disorders (Bantjes et al. 2023:217). Lazarus and Folkman’s (1987) Transactional Theory of Stress and Coping served as a theoretical framework for the study, highlighting the importance of understanding how nursing students cope with anxiety-related conditions, with an emphasis on positive coping strategies (Lazarus & Folkman 1987). Middleton (2020:14) argued that simply identifying and referring individuals is not enough to address the country’s increasing demand for mental health services.

This study developed supportive strategies to enable nursing students to manage their anxiety and to promote academic success and well-being. These strategies were guided by the World Health Organization’s (WHO 2018) steps of strategy development.

## Research methods and design

### Study design

This article is part of a larger two-phase descriptive phenomenological study. In phase 1, the researcher explored and described the lived experiences of nursing students and the perspectives of nurse educators regarding students’ anxieties, which were used as a foundation. The study setting was three Nursing Education Institutions (NEIs) in Gauteng Province, South Africa.

Undergraduate nursing students were purposively sampled based on anxiety-related experiences during training and were referred to the counselling department and consulted for support. Nurse educators were purposely sampled based on their experience teaching nursing students with anxiety. All nursing student participants were above 18 years and had been supported by the counselling department from January 2019 to December 2020.

Eight nursing students and four nurse educators who work in the counselling department were interviewed individually, and four focus group discussions with five nurse educators in each group were conducted on Microsoft Teams. The duration of the interviews were 30–60 min, and the focus group was about 90 min.

Data collection was conducted from May 2021 to September 2021. Data were analysed using Giorgi’s (2009) four-step method, where the entire set of transcripts was read to gain a holistic understanding of the data in order to identify the meaning units; the data were synthesised, and themes and sub-themes were identified.

### Ethical considerations

Ethical approval was obtained from the University of South Africa’s Health Research Ethics Committee (Reference 08039992_CREC_CHS_2021) and the Gauteng Department of Health (Reference number: 2021 03080). Permission to conduct the study was obtained from the head of each nursing education institution (Reference Number: 2021/04/002). Confidentiality was maintained throughout the presentation of findings and the development of strategies. Participants’ data were kept confidential, and no names were linked to participants’ data. Informed consent was obtained voluntarily after the participant understood the information about the phenomenon. Participants were not coerced to participate and had the right to change their minds after they had agreed. The researcher had arranged with the counselling staff to attend to the nursing students after the interviews.

## Results

In Seshabela and Shakwane (2024:4), the findings related to nurse educators highlighted the effects of anxiety-related conditions on the academic progress of nursing students. This article will focus on phase 2 of the study, which was promoting supportive strategies that will enable nursing students to manage their anxiety.

### Phase 2: Development of strategies to manage anxiety-related conditions

The researchers used the findings from data collection and analysis (empirical phase) to develop the strategies. The WHO (2018) five steps guided the researcher to develop strategies to manage anxiety-related conditions.

#### Step 1: Situational analysis

Conducting a situational analysis was the first step in building a better understanding of the current state of quality, the existing strengths and weaknesses of NEIs for quality improvement.

#### Step 2: Development of goals and priorities

Understanding the existing NEIs’ challenges from the situational analysis was crucial. The data collected allowed the researcher to formulate goals and priorities, focusing on the need to achieve in the management of anxiety-related conditions.

#### Step 3: Improvement methods and interventions

In the study, provisional strategies were developed to address identified gaps and enhance the implementation of strategies.

#### Step 4: Defining quality

Strategies for the management of anxiety-related conditions were developed to ensure that anxiety-related conditions were identified at the pre-selection of nursing students’ intake for prompt intervention and for continuity of care to provide a healthy mind among nursing students on completion.

#### Step 5: Stakeholder involvement

The researcher engaged with stakeholders regarding the proposed strategies. The stakeholders acknowledged the strategies, agreed with the researcher on the identified priorities, formulated new goals and identified gaps. The steps to be discussed on how these steps were applied to the development of strategies after the integrated findings.

### The integrated findings from the empirical study

Integrated findings from the two participant groups were: understanding of anxiety-related conditions, influences of anxiety-related conditions, responses to anxiety-related conditions and management of anxiety-related conditions among nursing students.

#### Understanding of anxiety-related conditions

Participants described their understanding of anxiety-related conditions as an inability to cope with stressful situations, an inability to make decisions, overwhelming feelings, uncertainty, fearfulness, a sense of powerlessness, apprehension, worry and uneasiness. Nursing students reported finding it difficult to cope with their academic studies when anxious.

#### Influences of anxiety-related conditions among nursing students

Participants highlighted the influence of anxiety-related conditions as those stemming from various sources, which are academic pressure within NEIs, as the programme entails intense theoretical and clinical modules, examinations and tests, including emotional and physical demands of caring for sick patients.

The lack of a supportive environment was another key influence, particularly in clinical settings. Nursing students often face negative attitudes from clinical staff and a lack of mentors, making it difficult for them to build the skills and confidence needed to care for patients effectively. Some nursing students lack discipline, so they team up with friends and use peer pressure, enjoying their freedom while neglecting their academic work, hence poor performance.

Some nursing students have chronic medical conditions that require regular follow-up, appointments, tests and medications. Nursing students with chronic medical conditions expressed their frustrations when they had to juggle medical appointments with academic load. Social responsibilities also contributed to nursing students’ anxiety as some had to care for family members, including spouses, children and siblings. Some needed to share their bursary money to support their families financially. Some come from dysfunctional families with abusive parents, which further increases their anxiety levels.

#### Responses to anxiety-related conditions

Nursing students experience physical and emotional responses when anxious. Physical manifestations such as sweaty hands, breathlessness, headache, fatigue and disturbed sleep. Emotional responses are irritability, worry, decreased concentration, fear, suicidal ideas and powerlessness.

#### Management of anxiety-related conditions among nursing students

Participants used various constructive methods to manage anxiety-related conditions, including deep breathing exercises, listening to music and meditation. Time management skills, self-awareness, building self-esteem, participating in group discussions and practising stress management techniques were also used. Singing, cleaning, praying, meditation and doing other things assist nursing students in coping, as it takes their minds off the anxiety. Studying timetables and a range of supportive techniques helps to reduce anxiety-related conditions among nursing students.

The availability of support from relevant professionals such as nurse educators, psychologists, pastors and peer mentors was emphasised as a crucial factor in helping students cope with anxiety. Figure 1 illustrates the summary of integrated findings from the empirical phase.

**FIGURE 1:**
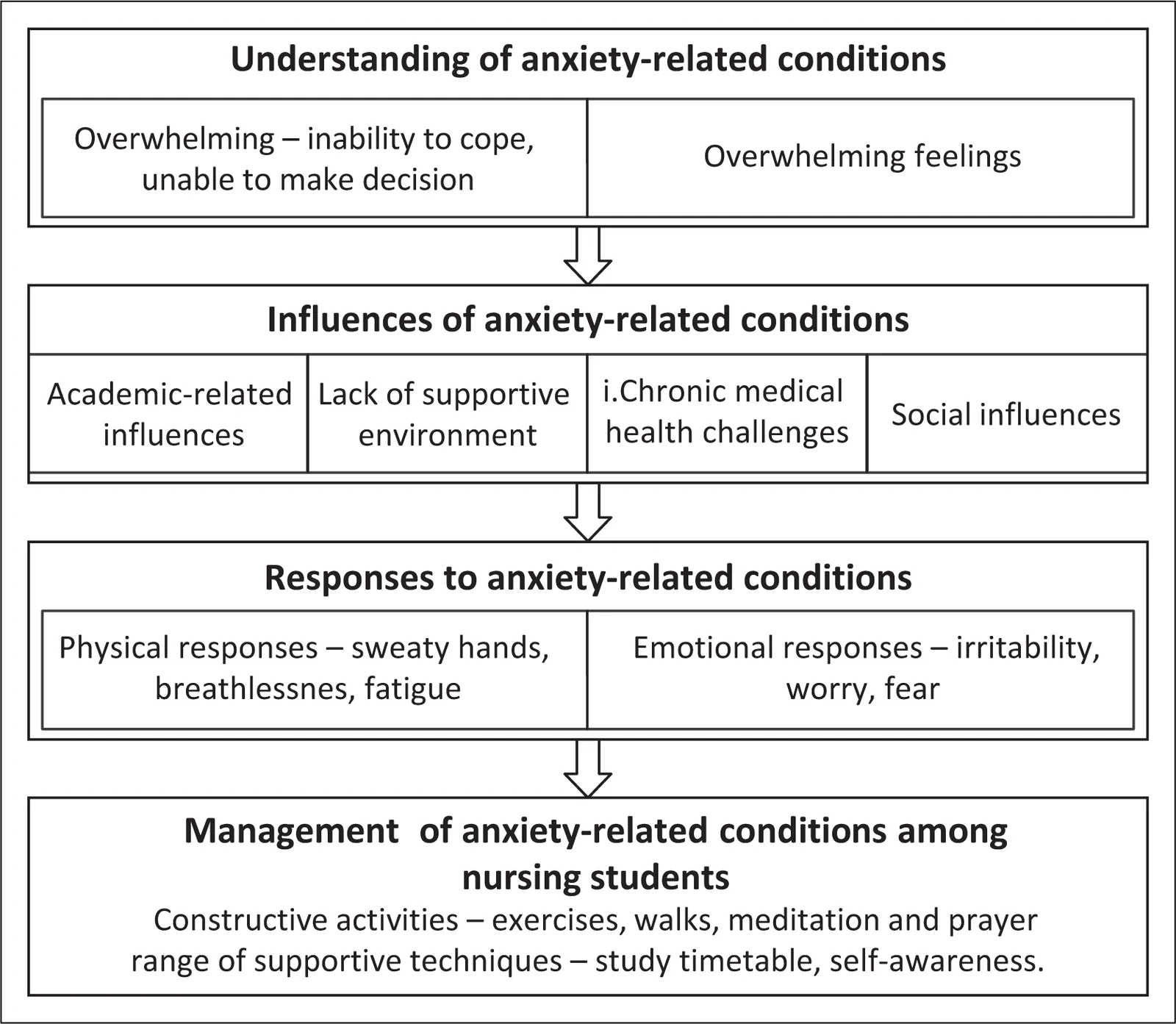
A summary of the main findings from phase 1.

### Presentation of developed strategies

Strategies involve the creation of unique and valuable positions that utilise different sets of activities (Cory 2024:4). The integrated findings from the empirical phase of the study were used to develop strategies to manage anxiety-related conditions among nursing students. The WHO’s (2018) strategy development steps were utilised in the Development of Strategies. The steps are briefly explained under the above methodology. Each step demonstrates the integration of the methods employed in developing strategies for managing anxiety-related conditions and was incorporated with the theory of Lazarus and Folkman’s (1987) Transactional Stress and Coping Theory. The WHO’s (2018) steps of strategy development are discussed individually below.

#### Step 1: Situational analysis: Reviewing the environment, personnel and procedures

The first step in developing the strategy for managing anxiety-related conditions among students was a situational analysis. This approach mapped key human and non-human elements, emphasising relationships and social factors. The researcher analysed the physical environment, personnel and procedures to gain a better understanding of the current state of the existing strengths and challenges of NEIs in the management of anxiety-related conditions. Using Lazarus and Folkman’s (1987) Transactional Theory of Stress and Coping, the researcher assessed mental health influences and triggers. A survey tool was utilised to evaluate the existing management mechanisms, consulting rooms, counsellors’ qualifications and additional training and experiences. Additionally, data on the number of nursing students referred in 2019 and 2020, as well as the reason for referrals, were reviewed.

The findings from the situational analysis of NEIs were as follows: In NEI – 001, three counsellors and soundproof consulting rooms were available. Seven nursing students consulted, including one male, according to records. Level 3. R.425 were referred to a psychologist, and six females (level 4, R.425) were treated in the counselling department (2019–2020). The records showed 40 students had academic issues, 30 had personal problems and one was referred to a psychologist. Academic support included study schedules and counselling sessions.

Nursing education institution – 002 had two counsellors and two non-soundproof consulting rooms. From 2019 to 2020, 14 students had academic issues, two had personal problems, one had chronic medical challenges and three had psychological issues, with no psychological referrals. The counsellors provided study schedules during theoretical blocks. The R.683 nursing programme met the inclusion criteria according to the records but was unavailable as its programme was phased out.

Nursing education institution – 003 lacked a counselling department, relying on affiliated psychologists. No situational analysis was conducted, and no clear guidelines existed for supporting nursing students with anxiety-related conditions. All counsellors in NEIs had no special training.

#### Step 2: Identification of gaps and development of priorities and goals

The future of the new NEIs’ curriculum programme is uncertain because of congested academic activities and a lack of recreational facilities for relaxation to alleviate anxiety. Situational analysis in two NEIs revealed that policies and guidelines neglect mental health issues, providing no direction to counsellors or nurse educators on the management of anxiety-related conditions. Academic policies do not address anxiety, highlighting their disregard for students’ mental health. Medical surveillance during pre-selection identifies support needs but only includes physical assessments, excluding mental health evaluations.

Stakeholders acknowledged that anxiety-related conditions are neither assessed nor managed at any level, leaving them unaddressed pre-selection and orientation. Early identification and intervention could improve students’ mental well-being and academic achievement and reduce long-term psychological problems (Coetzer, Bold & Van der Mark 2022:2). A summary of all Gauteng Colleges of Nursing policies highlights that these gaps can be found in Table 1.

**TABLE 1:** Gauteng College of Nursing policies related to mental health.

Name of the policy	Number	Purpose	Gaps
The student monitoring and progression policy are the policy for the nursing student who has been identified as making unsatisfactory progress and are at risk of not making progress.	(27/GCON/2018)	The purpose of this policy is to enable the nursing education institution to structure the flow of the programme and create instructor-paced learning for the nursing student.	There are gaps in all policies for these priorities. Understanding anxiety-related conditions, responses displayed by nursing students, influences and approaches are all neglected in policy number 27/GCON/2018, (03/GCON/2018, 06/GCON/2018 and 2/6/1/2019.
Teaching and learning policy are the policy that serves as a guiding framework for decision-making regarding teaching and learning in nursing education institutions.	(03/GCON/2018	Support for non-active and at-risk students.	
Support for non-active and at-risk students	06/GCON/2018	The policy provides guidelines on measures to systematically identify and support non-active and at-risk students to optimise their personal and professional growth towards academic success.	
The referral to the counselling department form (the Gauteng Internal Guideline and Procedures (IGP) number	(2/6/1:2019)	The policy provides guidelines and procedures for access to services rendered by the counselling department to provide the framework to support the development of quality nursing students who can provide excellent healthcare services to the community.	

#### Development of priorities

Setting out priorities in this study was vital to addressing the gaps and needs. Priorities are needed to ensure the development of quality strategies that target anxiety (Wong et al. 2022:1). The researcher used these gaps and findings to establish priorities as outlined in Figure 1. The results highlight the need to prioritise mental health in nursing education as healthcare professionals face various stressors, like staff shortages, critical patients and the coronavirus disease 2019 (COVID-19) pandemic. Once priorities were developed, goals were set to establish the required results. Goal setting is the process through which individuals establish aims and objectives for their actions in managing intended outcomes (Bird, Swannb & Jackman 2024:2). The study’s goal, based on identified priorities, guides strategic implementation. According to WHO (2018), setting priorities and goals is key to strategy development.

**Priority 1: Understanding anxiety-related conditions:** Situational analysis gaps were identified and assisted in the formulation of priority 1. Table 2 explains priority 1, the identified gaps and goals to be achieved, followed by a discussion of priority 1.

**TABLE 2:** Priority 1, identified gaps and goals.

Priority	Gaps	Goals
Priority 1: Understanding anxiety-related conditions	Mental health issues were not incorporated into the Gauteng College of Nursing procedure manuals and policies. Awareness sessions for stakeholders on mental health issues are neglected. Nurse educators reported not being empowered to identify anxiety-related conditions among nursing students. Insufficient infrastructure to provide quality mental health care services in nursing education institutions (virtual counselling and diversity [age and culture])	Create awareness through orientation and in-service training. Awareness of anxiety-related conditions among stakeholders. Educate nursing students on self-awareness and self-monitoring

Priority 1, focused on enhancing understanding of anxiety-related conditions by raising awareness, providing education, and establishing adequate infrastructure to meet the diverse needs of students and ensure the delivery of quality support services.

Priority 1 was understanding anxiety-related conditions. Identified gaps included the absence of guidelines about mental health issues in NEIs, the lack of discussion during orientation and the exclusion of mental health in professional development for nurse educators. Additionally, there is insufficient infrastructure for the quality of mental health services within NEIs. Mental health impacts productivity and well-being, which can be increased by long shifts and a lack of support among nursing students, leading to poor academic success (Hussein, Hashish & Younes 2024:1). Middleton (2020:149) highlighted that cultural diversity influences health definitions and decision-making. Therefore, NEIs should review policies to prioritise mental health in nursing education. The findings in the table emphasise an age gap between nursing students, suggesting potential differences in approach.

**Priority 2: Assessment of the influences of anxiety-related conditions among nursing students:** Assessment of the influence of anxiety-related conditions among nursing students in priority 2 is important, aimed at educating nursing students on the influences of anxiety-related conditions and empowering nurse educators to assess these influences among students. This is to be achieved by closing this gap, that is, while physical assessments are conducted during the pre-selection period, mental health and professional development programmes often neglect mental health issues. Table 3 explains Priority 2, its gaps and goals, followed by a discussion of Priority 2.

**TABLE 3:** Priority 2, identified gaps and goals.

Priority	Gap	Goal
Priority 2: Assessments of the influences of anxiety-related conditions among nursing students	During the pre-selection of nursing students, during medical surveillance, physical assessments are done, but mental health issues are neglected. In the clinical professional development (CPD) programmes records, mental health issues are neglected.	Educating nursing students about the influences of anxiety-related conditions. Empower nurse educators in assessing the influence of anxiety-related conditions among nursing students.

Physical and mental health significantly affect the independence of individuals (Mahindru, Patil & Agrawal 2023:1). Nursing education has long been associated with anxiety, with students experiencing it throughout their training (Garcia-Gonzalez et al. 2021). Therefore, it is essential to educate nursing students and equip nurses to assess anxiety-related conditions. Academic pressure, combined with social issues such as trauma or physical illness, increases anxiety (Savitsky et al. 2020:2). A new environment, unfamiliar peers and a lack of support can be challenging. According to Lazarus and Folkman (1987), the relationship between an individual and their environment can influence communication and comfort levels. To address these challenges, the clinical setting must be prepared for nursing students’ placements. This includes assessing clinical facilities, providing them with the necessary information and documents, learning outcomes, as well as offering clinical mentors and ensuring that supportive clinical coordinators are available (Bester, Van Wyk & Maree 2024:2).

**Priority 3: Responses to anxiety-related conditions:** The researchers aimed to educate nursing students to recognise physical and emotional symptoms of anxiety and to empower nurse educators to effectively address anxiety-related conditions. Table 4 describes priority 3, identified gaps and goals. The table is followed by a priority 3 discussion.

**TABLE 4:** Priority 3, identified gaps and goals.

Priority	Gap	Goal
Priority 3: Responses to anxiety-related conditions	Mental health issues are not incorporated into the orientation programme for the newly appointed intake of nursing students. Workshops or in-service training are not scheduled for stakeholders in the NEIs.	Educate nursing students about self-awareness so that they are aware of the physical and emotional symptoms displayed when anxious. Empower counsellors and nurse educators about responses to anxiety-related conditions.

NEI, nursing education institution.

Mental health issues are currently not addressed in NEIs’ policies, and there are no workshops or training for nurse educators on how to respond. Also, the nature of anxiety-related disorders, which increase the risk of depression and substance use disorders, affected individuals are also at risk of suicidal thoughts and behaviours (WHO 2025). Mental health disorders are more prevalent in nursing students, making it crucial for students and nurse educators to be aware of anxiety symptoms and responses.

**Priority 4: Management of anxiety-related conditions among nursing students:** Management of anxiety-related conditions aimed at educating nursing students and stakeholders on the management of anxiety-related conditions. Table 5 describes priority 4, identified gaps and goals. The table is followed by a priority 4 discussion.

**TABLE 5:** Priority 4, identified gaps and goals.

Priority	Gap	Goal
Priority 4: Management of anxiety-related conditions among nursing students.	Orientation about available support and strategies to cope with anxiety-related conditions is not done in NEIs. No sufficient qualified counsellors to provide comprehensive care to ensure that nursing students are treated holistically in the NEIs.	Teach nurse educators, counsellors and nursing students about the strategies for managing anxiety-related conditions among nursing students.

NEI, nursing education institution.

The management of anxiety-related conditions is a priority for NEIs. However, there is a gap in the provision of sufficient orientation, support and strategies for nursing students to effectively cope with these conditions. Additionally, the availability of qualified counsellors who can provide comprehensive care to nursing students is limited in NEIs. Anxiety can be challenging to diagnose, as somatic symptoms often outweigh psychological ones. Understanding anxiety-related conditions helps differentiate between anxiety and somatic symptoms (Huang et al. 2021:3).

#### Step 3: Improvement methods and interventions

To implement strategic planning effectively, quality improvement interventions must be identified to address key priorities and achieve specific quality goals (WHO 2018). The theory for the study was the Theory of Stress and Coping by Lazarus and Folkman (Keltner & Steele 2023:317), which emphasises the role of cognitive and/or primary appraisal in evaluating stress levels and stressors. The theory suggests that stressors from the environment and responses to them can threaten an individual’s well-being. If the stress persists despite primary appraisals, secondary appraisals are utilised, leading to further management through referrals (Lazarus & Folkman 1987). Primary prevention occurs during primary appraisals, while secondary appraisals involve treatment. Effective management of anxiety-related conditions is essential for nursing students to succeed academically. The Theory of Stress and Coping by Lazarus and Folkman (1987) also emphasises the importance of secondary appraisals in dealing with ongoing issues and implementing improved interventions.

#### Step 4: Defining quality

The researchers developed strategies based on gaps identified from situational analysis. Priorities formulated from findings were established, and goals were written based on the desired outcomes. These strategies aim to increase awareness of anxiety-related conditions among nursing students, helping them manage distressing moments. They also intend to empower nurse educators with the knowledge and skills to assist students with anxiety-related conditions for quality improvements. Tentative strategies were developed and presented to stakeholders for their validation and input to enhance the quality of the methods and interventions for managing anxiety in nursing students. Also, their advice on monitoring and evaluations is very important for quality improvement and for the NEIs.

Effective monitoring and evaluation are crucial in determining the quality of strategies addressing anxiety-related issues (Mabizela & Zwane 2023:2). The WHO (2018:13) defines health quality by its effectiveness, efficiency, safety, individual-centred timeliness, equity and integration. The Transactional Theory of Stress and Coping by Lazarus and Folkman (1987) focuses on primary appraisals as the continuous evaluation of events based on their impact on well-being (Lazarus & Folkman 1987). The individual assesses their resources, skills and knowledge in coping with the situation (Lazarus & Folkman 1987). The study raises awareness of anxiety-related conditions among nursing students, their influences, responses and management. Nursing students are to be introduced to anxiety-related influences, responses and management. Students will be oriented to anxiety-related issues during their first year of training, with effective monitoring and evaluation tools used to enhance quality in managing anxiety in nursing education.

#### Step 5: Stakeholder engagement and validation

Engaging stakeholders was essential for the continuity and sustainability of care. The researcher shared the tentative strategies with stakeholders for feedback and refinement to address implementation gaps. Stakeholders were carefully selected based on their expertise with nursing students, including counsellors. Heads of Department, nurse educators and occupational health nurses from three NEIs, which were two public colleges and one university. Appointments were scheduled via email, with presentations held on Microsoft Teams, and in one institution, face-to-face meetings were conducted. Strategies were presented according to the gaps and priorities identified through the study’s themes and goals. The discussion was recorded for reference. Most stakeholders recognised the importance of strengthening mental health support in NEIs. The tentative strategies were seen as a valuable tool for promoting mental health stability in nursing students, thereby enhancing academic success.

## Strategies for managing anxiety-related conditions

The developing strategies to manage anxiety-related conditions among nursing students were guided by four key priorities identified from empirical findings of participants’ feedback. However, priorities two and three were closely related and were combined, resulting in three priorities. Strategies were then developed to address identified gaps, aligned with these priorities, and establish new objectives aimed at fostering mental resilience and promoting academic success among nursing students. This approach was informed by Lazarus and Folkman’s (1987) *Transactional Theory of Stress and Coping*, which provided a theoretical framework for understanding anxiety management. The process begins with cognitive or primary appraisals, where interventions such as awareness creation, empowerment and competency development are introduced. If anxiety persists despite these measures, a secondary appraisal follows, necessitating additional support and treatment. The proposed strategies were reviewed and endorsed by stakeholders who reached a consensus with the researcher on their relevance and applicability. Figure 2 presents the summary of the final strategies for managing anxiety-related conditions.

**FIGURE 2:**
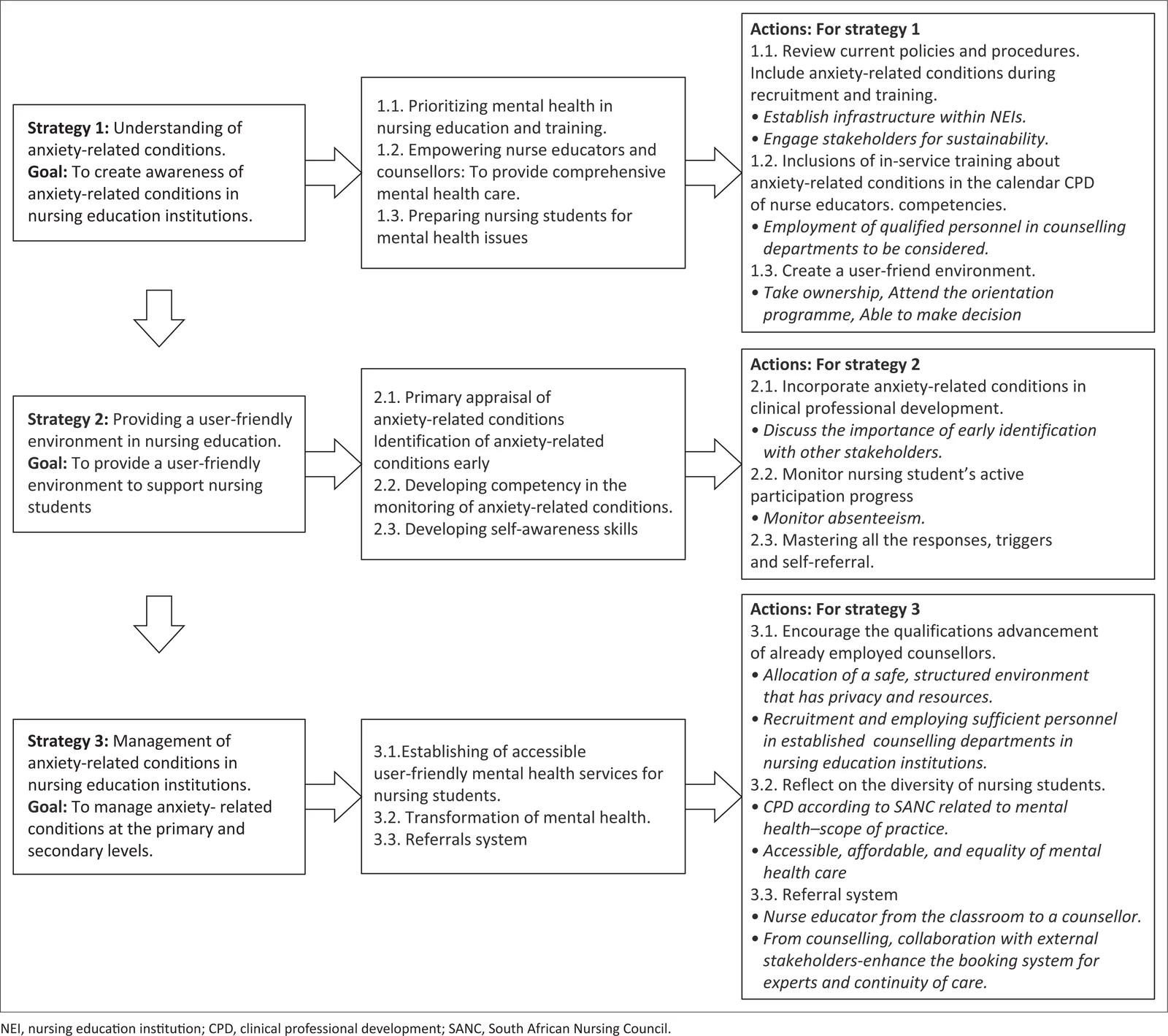
Supportive strategies for the management of anxiety-related conditions.

## Discussion of the supportive strategies

### Strategy 1: Understanding anxiety-related conditions

This is a foundational step in supporting nursing students, involving the improvement of understanding and recognition of anxiety-related conditions. This includes reviewing existing policies and procedures to ensure they address anxiety-related conditions, establishing support infrastructure within NEIs and offering in-service training and workshops. Anxiety disorders are associated with significant morbidity, including substance abuse, alcoholism and major depression with a high risk of suicide (Chand & Marwaha 2025:1). As noted by Naveen et al. (2024:2), effective intervention requires a comprehensive understanding of both the psychological and physiological components of anxiety. Additionally, Ratering, Van der Heijden and Martens (2024:1) highlight the importance of understanding how anxiety influences individuals’ mobility behaviour and opportunities, an essential consideration in efforts to promote inclusive practice. For nursing students, developing a thorough understanding of anxiety-related conditions is not only for their personal well-being and academic performance but also for their professional preparedness in addressing mental health needs in the clinical setting. Without this understanding, students experiencing anxiety may be more vulnerable to other mental health issues, which can adversely affect their academic success (Rani 2024:1). Moreover, unrecognised or misunderstood anxiety can manifest as impulsive behaviour and emotional instability, further disrupting the students’ learning process (WHO 2025).

### Strategy 2: Providing a user-friendly environment in nursing education

Creating a user-friendly environment within nursing education is essential for supporting students’ mental health and academic performance. This involves understanding students’ responses and triggers, prioritising early identification of anxiety-related conditions and monitoring nursing students’ progress and absenteeism. Mental health issues and associated stressors can significantly interfere with nursing students’ academic performance. Early identification and intervention can help minimise these risk factors (Hawamdeh et al. 2023:2). Identifying and understanding the unique challenges nursing students face in the learning environment allows nurse educators and stakeholders to implement targeted support strategies to promote both academic success and long-term professional sustainability (Ahmed et al. 2023:2).

Therefore, nursing education programmes must prioritise mental health assessments of nursing students. This includes equipping all nurse educators and counsellors with clear responsibilities and standardised tools embedded within institutional guidelines. The assessment tool should be integrated with the internal guidelines and procedures and used consistently during the pre-assessment stage and when needed. Conducting assessments during these stages can facilitate timely support and ensure continuity of care for nursing students experiencing anxiety-related conditions and immediate treatment. Stress, physical health and environmental factors can contribute to the development of anxiety symptoms and play a role in intensifying anxiety experiences (Adwas, Jbireal & Azab 2019:2). Adjustment to a new lifestyle at a higher education institution necessitates flexibility and discipline; failure to adapt effectively to a new environment can increase vulnerability to anxiety (Mofatteh 2020:9). Assessment periods are often reported as high-stress times because of the significant academic demands faced by nursing students (Stevens, Nussbaum & Blake 2019:17). Nursing students with chronic medical conditions are particularly at high risk of developing anxiety disorders, which may negatively impact both their academic and health outcomes (Cobham et al. 2020:1). Emotional responses often stem from the accumulation of unresolved emotional memories over time, whereby an individual may interpret experience to find meaning in their trauma and open the door to healing and growth (Helio, Adonis & Guilherme 2022:7).

### Strategy 3: Management of anxiety-related conditions

Effective management of anxiety-related conditions within nursing education requires a multi-pronged approach that includes professional development, institutional collaboration and holistic support for students. Institutions should encourage the qualification advancement of employed counsellors and ensure the recruitment of sufficient mental health personnel. These efforts should be complemented by a recognition of the diverse backgrounds and mental health challenges faced by nursing students, which will need to be supported by a transparent and accessible referral system. There is increasing recognition of the important role of physical activity, which plays in the treatment of mental disorders, including anxiety (Carter et al. 2021:1). Nursing students employ a variety of coping strategies to manage anxiety, including venting, humour, religious practices and seeking social support (Savitsky et al. 2020:1). However, without structured support, these strategies may not be sufficient.

Nursing education institutions should actively collaborate with both internal and external stakeholders to provide support for nursing students dealing with anxiety (Skeen et al. 2022:3). Nurse educators should develop programmes aimed at assisting and supporting nursing students to cope with anxiety in enhancing their coping skills (Onieva-Zafra et al. 2020:2). Management strategies should adopt a holistic approach incorporating balanced nutrition, regular physical activities, sleep hygiene and opportunities for social interaction, which contribute to overall psychological resilience (Chi et al. 2021:2). These elements are foundational to overall mental wellness and should be integrated into nursing students’ support initiatives. To achieve this, NEIs must establish internal policies and structured guidelines that support nurse educators, counsellors and stakeholders in addressing anxiety. These frameworks should include training, orientation programmes and ongoing support systems, all aligned with institutional and mental health objectives. Such structures not only elevate the quality of care but also ensure efficient implementation and create efficient referral pathways for nursing students in need. As for Sovold et al. (2021:1) highlight, prioritising the delivery of mental health care services to prevent the accumulation of stress and foster academic success.

## Conclusion

The purpose of the study was to develop and validate supportive strategies that will enable nursing students to manage their anxiety during training. Comprehensive and supportive strategies are essential for addressing anxiety-related conditions among nursing students effectively. The active involvement of nurse educators is crucial in empowering both nursing students and stakeholders with the necessary knowledge, skills and resources to effectively manage anxiety. These strategies emphasise the importance of creating a supportive and inclusive educational environment, promoting positive attitudes towards mental health. Also, establishing clear collaborative referral pathways with mental health professionals. These measures enable early identification and prompt intervention, ensuring continuity of care and supporting academic success. It is therefore strongly recommended that NEIs adopt, promote and implement these strategies to enhance mental health support, academic success and long-term professional sustainability for nursing students.

### Recommendations

Nursing education institutions should align their mental health policies and procedures with the nursing students’ curriculum. These policies should be continuously reviewed and updated based on feedback from nursing students and mental health professionals to ensure they provide ongoing support.

### Limitations

Semi-structured interviews were conducted telephonically from May 2021 to September 2021, because of the country being in COVID-19 pandemic alert level 3 lockdown restrictions. The study was limited to a qualitative approach, which was carried out at three selected NEIs. Strategies have not yet been implemented; therefore, the study recommends their implementation to enable evaluation of their effectiveness.

## References

[CIT0001] Adwas, A.A., Jbireal, J.M. & Azab, A.E., 2019, ‘Anxiety: Insight into signs, symptoms, aetiology, pathophysiology, and treatment’, *East African Scholars Journal of Medical Sciences* 2(10), 580–591.

[CIT0002] Ahmed, F.A., Alrashidi, N., Mohamed, R.A., Asiril, A., Ali, A.A., Aly, K.H. et al., 2023, ‘Satisfaction and anxiety level during clinical training among nursing students’, *BMC Nursing* 22, 187. 10.1186/s12912-023-01352-3 PMC10239170 37268973

[CIT0003] Bantjes, P.J., Kessler, M., Lochner, C., Breet, E., Bawa, A., Roos, J. et al., 2023, ‘The mental health of university students in South Africa: Results of the national student survey’, *Journal of Affective Disorders* 321, 217–226. 10.1016/j.jad.2022.10.044 36349649

[CIT0004] Bester, E., Van Wyk, N.C. & Maree, C., 2024, ‘Development of collaboration guidelines for nursing education and related healthcare services’, *Health SA Gesondheid* 29, 2496. 10.4102/hsag.v29i0.2496 PMC10913140 38445031

[CIT0005] Bird, M.D., Swannb, C. & Jackman, P.C., 2024, ‘The what, why, and how of goal setting: A review of the goal-setting process in applied sport psychology practice’, *Journal of Applied Sport Psychology* 36(1), 75–97. 10.1080/10413200.2023.2185699

[CIT0006] Carter, T., Pascoe, M., Bastounis, A., Morres, I.D., Callaghan, P. & Parker, A., 2021, ‘The effect of physical activity on anxiety in children and young people: A systematic, and meta-analysis’, *Journal of Affective Disorders* 285, 10–21. 10.1016/j.jad.2021.02.026 33618056

[CIT0007] Chand, S.P. & Marwaha, R., 2025, ‘Anxiety’, in *StatPearls*, StatPearls Publishing, Treasure Island, FL, viewed n.d., from https://www.ncbi.nlm.nih.gov/books/NBK470361.

[CIT0008] Chi, X., Liang, K., Chen, S-T., Huang, Q., Huang, L., Yu, Q. et al., 2021, ‘Mental health problems among Chinese adolescents during COVID-19: The importance of nutrition and physical activity’, *International Journal of Clinical and Health Psychology* 21(1), 100218. 10.1016/j.ijchp.2020.100218 PMC7759093 33391373

[CIT0009] Cobham, V.E., Hickling, A., Kimball, H., Thomas, H.J., Scott, J.G. & Middeldorp, C.M., 2020, ‘Systematic review. Anxiety in children and adolescents with chronic medical conditions’, *Journal of the American Academy of Child and Adolescent Psychiatry* 59(5), 595–618. 10.1016/j.jaac.2019.10.010 31676391

[CIT0010] Coetzer, J.A., Bold, A. & Van der Mark, E., 2022, ‘Exploring mental health interventions for youth in Southern Africa: A rapid review’, *Acta Psychologica* 229, 103699. 10.1016/j.actpsy.2022.103699 35952512

[CIT0011] Colizzi, M., Lusalvia, A. & Ruggeri, M., 2020, ‘Prevention and early intervention in youth mental health is it time for a multidisciplinary and trans-diagnostic model of care?’, *International Journal of Mental Health Systems* 14, 23. 10.1186/s13033-020-00356-9 PMC7092613 32226481

[CIT0012] Cory, K., 2024, ‘Revisiting “What is strategy?” and why it matters’, *Strategy & Leadership* 52(1), 1–6. 10.1108/SL-07-2023-0077

[CIT0013] Garcia-Gonzalez, J., Ruqiong, W., Alarcon-Rodriguez, R., Reguena-Muller, M., Ding, C. & Ventura-Miranda, M.I., 2021, ‘Analysis of anxiety levels of nursing students because of e-learning during the COVID-19 pandemic’, *Healthcare* 9(3), 252. 10.3390/healthcare9030252 PMC8001948 33804344

[CIT0014] Giorgi, A., 2009, *The descriptive phenomenological method in psychology: A modified Husserlian approach*, University Press, Duquesne, PA.

[CIT0015] Hawamdeh, S., Moussa, F.L., Al-Rawashdeh, S., Hawamdih, S.A. & Moussa, M.L., 2023, ‘Illness anxiety disorder and distress among female medical and nursing students’, *Clinical Practice and Epidemiology in Mental Health* 19, e17450179277976. 10.2174/0117450179277976231115070100 PMC11037515 38655550

[CIT0016] Helio, G.R.N., Adonis, T. & Guilherme, M., 2022, ‘Phenomenological description of PTSD through a case’, *Revista Psicopatologia Fenomenológica Contemporânea* 10(1), 39–75. 10.37067/rpfc.v10i1.1072

[CIT0017] Huang, Y., Zhou, Y., Zeng, L., Yang, J., Li, Y., Song, X. et al., 2021, ‘Differences in the Association of Anxiety, Insomnia and Somatic symptoms between medical staff and the general population during an outbreak of COVID-19’, *Neuropsychiatric Disease and Treatment* 17, 1907–1915. 10.2147/NDT.S300719 PMC8214107 34163164

[CIT0018] Hussein, A.H.M., Hashish, E.A.A. & Younes, B.M., 2024, ‘The relationship between nurses’ psychological well-being and their work productivity loss: A descriptive correlational study’, *Sage Open Nursing* 10, 1–14. 10.1177/23779608241285400 PMC11456208 39371426

[CIT0019] Jacobs, E., Scrooby, B. & Du Preez, A., 2019, ‘Experiences of student nurses regarding the bursary system in KwaZulu-Natal province, South Africa’, *Health SA Gesondheid* 24, a1103. 10.4102/hsag.v24i0.1103 PMC6917381 31934414

[CIT0020] Jamil, H., Alakkari, M., Al-Muhammed, M.S., Alsayid, M. & Jandali, O.A., 2022, ‘The impact of anxiety and depression on academics: A cross-sectional study among medical students in Syria’, *Avicenna Journal of Medicine* 12(3), 111–119. 10.1055/s-0042-1755181 PMC9458347 36092387

[CIT0021] Keltner, N.I. & Steele, D., 2023, *Psychiatric nursing*, 9th edn., Elsevier, St Louis, MO.

[CIT0022] Lazarus, R.S. & Folkman, S., 1987, ‘Transactional theory and research on emotions and coping’, *European Journal of Personality* 1(3), 141–169. 10.1002/per.2410010304

[CIT0023] Mabizela, H. & Zwane, Z., 2023, ‘Monitoring and evaluation as a critical approach to enhance the performance of local government: South Africa’, *International Journal of Research in Business and Social Science* 12(7), 74–84. 10.20525/ijrbs.v12i7.2746

[CIT0024] Macintyre, M.M., Zare, M. & Williams, M., 2023, ‘Anxiety-related disorders in the context of racism’, *Current Psychiatry Reports* 25(2), 31–43. 10.1007/s11920-022-01408-2. PMC9841144 36645562

[CIT0025] Mahindru, A., Patil, P. & Agrawal, V., 2023, ‘Role of physical activity on mental health and well-being: A review’, *Cureus* 15(1), e33475. 10.7759/cureus.33475 PMC9902068 36756008

[CIT0026] Manana, M., Ntuli, S.T., Mokwena, K. & Maaga, K., 2023, ‘Prevalence and risk factors for anxiety symptoms among student nurses in Gauteng Province of South Africa’, *Behavioral Sciences (Basel)* 13(8), 630. 10.3390/bs13080630 PMC10452000 37622771

[CIT0027] Middleton, L., 2020, *Mental health nursing: A South African perspective*, 7th edn., Juta, Cape Town.

[CIT0028] Mofatteh, M., 2020, ‘Risk factors associated with stress, anxiety, and depression among university undergraduate students’, *AIMS Public Health* 8(1), 36–65. 10.3934/publichealth.2021004 PMC7870388 33575406

[CIT0029] Mudaly, P.D., 2023, ‘Challenges of first year students nurses in an undergraduate nursing higher education institution programme, South Africa’, *IOSR Journal of Nursing and Health Science* 12(3), 13–20.

[CIT0030] Naveen, V., Felic, S., Aswin, A.V. & Arul, P.K.C., 2024, ‘A review of current understanding, clinical manifestations, and therapeutic approaches to anxiety disorders’, *Journal of Pharma Insights and Research* 2(5), 173–181. 10.69613/xy1yek24

[CIT0031] Onieva-Zafra, M.D., Fernandez-Munoz, J.J., Fernandez-Martinez, E., Garcia-Sanchez, F.J., Abreu-Sanchez, A. & Parra-Fernandez, L., 2020, ‘Anxiety, perceived stress and coping strategies in nursing: A cross-sectional, correlational, descriptive study’, *BMC Medical Education* 20, 370. 10.1186/s12909-020-02294-z PMC7574568 33081751

[CIT0032] Otim, M., Marzouqi, M.A., Sabu, M., Damai, N. & Al-Harbaw, S., 2021, ‘Prevalence of generalised anxiety disorders among clinical training students at the University of Sharjah’, *Journal of Multidisciplinary Healthcare* 14, 1863–1872. 10.2147/JMDH.S317828 PMC8286110 34285504

[CIT0033] Rani, S., 2024, ‘Studying the impact of anxiety, stress, and emotion on academic performance: A systematic review’, *Journal of Social, Humanity and Education (JSHE)* 5(2), 131–141. 10.35912/jshe.v5i2.2437

[CIT0034] Ratering, C., Van der Heijden, R. & Martens, K., 2024, ‘Moving around with an anxiety disorder’, *Transportation Research Part F: Traffic Psychology and Behaviour* 100, 493–506. 10.1016/j.trf.12.005

[CIT0035] Savitsky, B., Findling, Y., Ereli, A. & Hendel, T., 2020, ‘Anxiety and coping strategies among nursing students during the COVID-19 pandemic’, *Nurse Education in Practice* 46, 102809. 10.1016/j.nepr.2020.102809 PMC7264940 32679465

[CIT0036] Seshabela, H.D. & Shakwane, S., 2024, ‘Impact of anxiety-related conditions on nursing students’ academic excellence’, *Curationis* 47(1), e1–e10. 10.4102/CURATIONIS.v47i1.2571 PMC11538042 39494647

[CIT0037] Skeen, S., Gemmell, K., Du Toit, S., Mawoyo, T., Bantjes, J., Kara, T. et al., 2022, ‘The role of educational institutions in promoting and protecting mental health across childhood, adolescence, and youth’, in M. Tomlinson, S. Kleintjes & L. Lake (eds.), *South African Child Gauge 2021/2022: Child and adolescent mental health*, pp. 86–98, Children’s Institute, University of Cape Town, viewed n.d., from https://ci.uct.ac.za/sites/default/files/content_migration/health_uct_ac_za/533/files/Child%2520Gauge%25202021_110822.pdf.

[CIT0038] South African Nursing Council, 2005, *Nursing Act, no.33, 2005*, Government Printer, Pretoria.

[CIT0039] Sovold, L.E., Naslund, J.A., Kousoulis, A.A., Saxena, S., Qoronfleh, M.W., Grobler, C. et al., 2021, ‘Prioritising the mental health and well-being of healthcare workers: An urgent global public health priority’, *Frontiers in Public Health* 9, 679397. 10.3389/fpubh.2021.679397 PMC8137852 34026720

[CIT0040] Stein, D.J., Craske, M.G., Rothbaum, B.O., Chamberlain, S.R., Fineberg, N.A., Choi, K.W. et al., 2021, ‘The clinical characterisation of the adult patient with an anxiety or related disorder aimed at personalisation of management’, *World Psychiatry* 20(3), 336–356. 10.1002/wps.20919 PMC8429350 34505377

[CIT0041] Stevens, R., Nussbaum, R. & Blake, T., 2019, *Evaluating state anxiety levels in nursing students*, Williams Honours College, Honours Research Project, viewed n.d., from https://ideaexchange.uakron.edu/honors_research_projects/815.

[CIT0042] Wong, E.C., Maher, A.R., Motala, A., Ross, R., Akinniranye, O., Larkin, J. et al., 2022, ‘Methods for identifying health research gaps, needs, and priorities. A scoping review’, *Journal of General Internal Medicine* 37(1), 198–205. 10.1007/s11606-021-07064-1. PMC8738821 34748098

[CIT0043] World Health Organization (WHO), 2018, *Handbook for national quality policy and strategy: A practical approach for developing policy and strategy to improve the quality of care*, viewed n.d., from https://www.who.int/publications/i/item/9789241565561.

[CIT0044] World Health Organization (WHO), 2025, *Anxiety disorders*, viewed n.d., from https://www.who.int/news-room/fact-sheets/detail/anxiety-disorders.

[CIT0045] Xi, Y., 2020, ‘Anxiety: A concept analysis’, *Frontiers of Nursing* 7(1), 9–12. 10.2478/fon-2020-0008

